# Comprehensive geriatric assessment for chronic obstructive pulmonary disease

**DOI:** 10.1097/JS9.0000000000003181

**Published:** 2025-08-20

**Authors:** Chunmei Shao, Yuan Liu, Meiju Yang

**Affiliations:** a Nursing Department, First People’s Hospital of Shangqiu, Henan, China; b Medical Department, First People’s Hospital of Shangqiu, Henan, China; c Department of Geriatrics, First People’s Hospital of Shangqiu, Henan, China

*Dear Editor,*

Chronic obstructive pulmonary disease (COPD) remains a leading cause of morbidity and mortality worldwide, disproportionately affecting older adults. As global ageing accelerates, with 21% of the world’s population projected to be over 60 years old by 2050, the imperative for a more holistic approach to elderly COPD care becomes undeniable[1]. Conventional clinical assessments, while effective in diagnosing airflow limitation and managing exacerbations, often overlook the multidimensional needs of older patients. Multimorbidity, cognitive decline, frailty, and polypharmacy complicate care delivery in this population, necessitating a more integrated and individualized framework. Comprehensive geriatric assessment (CGA) has emerged as a cornerstone of geriatric medicine, demonstrating consistent benefits across clinical settings[2]. This correspondence is compliant with the TITAN Guidelines 2025—governing declaration and use of AI[3].

In patients with COPD, CGA has been associated with reduced anxiety and depression, extended symptom relief, and decreased risk of complications such as cardiovascular disease, osteoporosis, and mental disorders[4]. Importantly, CGA facilitates identification and management of geriatric syndromes, which in turn may lower the risk of COPD exacerbations and hospitalizations[4].

Despite these compelling benefits, widespread implementation of CGA remains limited due to its resource-intensive nature, workforce shortages, and operational hurdles[5]. Here, digital innovation presents a transformative solution. Mobile health applications, wearable sensors, and web-based pulmonary rehabilitation programs can enhance symptom monitoring and patient engagement. Telemedicine, particularly when augmented with remote monitoring, has shown promise in reducing hospital readmissions. Meanwhile, artificial intelligence enables earlier detection of emphysema and structural lung changes, which could optimize drug management and enhance psychological support through chatbots[6]. A synergistic, digitally enabled CGA model can leverage technology to extend the reach and efficiency of geriatric care for COPD patients. This approach has the potential to narrow the persistent “know-do gap,” particularly in settings with limited access to geriatric expertise.

To advance this vision, research must now focus on implementation science-developing scalable models, validating digital tools in frail populations, and embedding CGA within policy and reimbursement frameworks. Only through such efforts can we ensure that COPD care for older adults is not only clinically effective, operationally feasible, and aligned with the evolving needs of ageing populations.

## Data Availability

All data are included in the article.

## References

[1] Liao K Yang D Jin L. Bidirectional mendelian randomization and single-cell sequencing reveal t cell-mediated causal links between COPD and lung adenocarcinoma. Int J Surg 2025;111:4528–38. 40676822 10.1097/JS9.0000000000002448

[2] Witt LJ Wroblewski KE Pinto JM. Beyond the lung: geriatric conditions afflict community-dwelling older adults with self-reported chronic obstructive pulmonary disease. Front Med 2022;9:814606. 10.3389/fmed.2022.814606 PMC8884078 35237627

[3] Agha RA Mathew G Rashid R. Transparency In The reporting of Artificial INtelligence – the TITAN guideline. Prem J Sci 2025;10:100083.

[4] Polidori MC, and Roller-Wirnsberger RE. Chances and challenges of comprehensive geriatric assessment training for healthcare providers. Geriatric Care 2018;4:7853.

[5] Sum G Nicholas SO Nai ZL Ding YY Tan WS. Health outcomes and implementation barriers and facilitators of comprehensive geriatric assessment in community settings: a systematic integrative review [PROSPERO registration no.: CRD42021229953]. BMC Geriatr 2022;22:379. 35488198 10.1186/s12877-022-03024-4 PMC9052611

[6] Zhuang M Hassan II Ahmad WMA. Effectiveness of Digital Health Interventions for Chronic Obstructive Pulmonary Disease: systematic Review and Meta-Analysis. J Med Internet Res 2025;27:e76323. 40418567 10.2196/76323 PMC12149779

